# Regulation of DNA Repair Mechanisms: How the Chromatin Environment Regulates the DNA Damage Response

**DOI:** 10.3390/ijms18081715

**Published:** 2017-08-05

**Authors:** Jens Stadler, Holger Richly

**Affiliations:** 1 Laboratory of Molecular Epigenetics, Institute of Molecular Biology (IMB), Ackermannweg 4, 55128 Mainz, Germany; j.stadler@imb-mainz.de; 2 Faculty of Biology, Johannes Gutenberg University, 55099 Mainz, Germany

**Keywords:** DNA repair, chromatin, histone marks, ubiquitin, nucleotide excision repair (NER), DSB repair

## Abstract

Cellular DNA is constantly challenged by damage-inducing factors derived from exogenous or endogenous sources. In order to maintain genome stability and integrity, cells have evolved a wide variety of DNA repair pathways which counteract different types of DNA lesions, also referred to as the DNA damage response (DDR). However, DNA in eukaryotes is highly organized and compacted into chromatin representing major constraints for all cellular pathways, including DNA repair pathways, which require DNA as their substrate. Therefore, the chromatin configuration surrounding the lesion site undergoes dramatic remodeling to facilitate access of DNA repair factors and subsequent removal of the DNA lesion. In this review, we focus on the question of how the cellular DNA repair pathways overcome the chromatin barrier, how the chromatin environment is rearranged to facilitate efficient DNA repair, which proteins mediate this re-organization process and, consequently, how the altered chromatin landscape is involved in the regulation of DNA damage responses.

## 1. Introduction

Genome integrity is permanently challenged by both exogenous and endogenous factors. In eukaryotes, damaged DNA is recognized and repaired by a variety of complex cellular pathways, which are referred to as the DNA damage response (DDR) [1,2]. The DNA damage response is characterized by the well-timed recruitment and removal of specific DNA repair factors and ancillary proteins, which mediate the recognition, verification, and repair of the lesion. However, DNA repair in eukaryotes occurs not simply on “naked” DNA. Eukaryotic genomes are organized and compacted into chromatin established by the essential building block, the nucleosome, which is generated from an octamer of the four core histones (H2A, H2B, H3, H4) around which the DNA is wrapped. Additionally, the mammalian genome is organized in specific chromatin structures that are characterized by signatures of histone and DNA modifications, specific histone variants, chromatin-associated factors, and nucleosome occupancy [3]. Thus, all cellular pathways, including the DNA damage response, which rely on DNA as their substrate, have to respond to and overcome this major constraint limiting the access of DNA repair factors, which is crucial for efficient recognition and removal of DNA lesions [4,5]. The importance of chromatin rearrangements and chromatin dynamics during the process of DNA repair was first indicated by nuclease digestion assays of damaged DNA in UV-C-irradiated fibroblasts in which replication was blocked by hydroxyurea treatment. Chromatin regions with active nucleotide excision repair (NER) were more prone to be digested by micrococcal nuclease treatment indicating that these regions have been rearranged to a more relaxed state and are, thus, more sensitive to nuclease treatment compared to compacted chromatin [6,7].

Another breakthrough regarding the question of how cells recognize and counteract DNA lesions in the context of chromatin, and how the chromatin landscape is altered to facilitate efficient DNA repair, emerged when human fibroblasts were pulse-labelled with (3H) deoxy-thymidine directly after UV irradiation and transferred into nonradioactive medium for recovery for different time points [7]. Interestingly, it was observed that the sensitivity of the incorporated nucleotides towards nuclease digestion decreased progressively depending on the time of recovery after UV irradiation and pulse-labelling and that, after a certain time of recovery, the original level of nuclease resistance was restored in the repaired DNA regions [7]. These findings built the first principles of the so-called “access-repair-restore” (ARR) model which highlights the frame conditions for the dynamic chromatin environment during DNA repair processes [5]. This model summarizes the transient de-condensation of chromatin structures to facilitate recognition and repair of DNA lesions. After the DNA lesion is removed, chromatin is reorganized and compacted to its original state. However, recent data also points out that new histones can be deposited during the restore section illustrating that the epigenetic memory allows some degree of plasticity [8]. Chromatin relaxation, as well as its re-condensation after removal of the DNA lesion, is a highly-regulated, ATP-consuming process, which requires the action of so-called chromatin remodeling complexes. Chromatin remodelers can be classified into four subfamilies depending on their domain organization: the imitation switch (ISWI), the chromodomain DNA helicase-binding (CHD), the switch/sucrose non-fermentable (SWI/SNF), and the INO80 subfamily. Furthermore, the four subfamilies can also be separated on a functional level since the different subfamilies preferentially catalyze specific functions. The SWI/SNF family of remodelers primarily conducts functions related to chromatin accessibility, which includes unwrapping of DNA coiled around nucleosomes, nucleosome re-positioning by the sliding of nucleosomes along the DNA, and partial (e.g., H2A-H2B dimer) or complete nucleosome eviction. In particular, access remodelers of the SWI/SNF subfamily can gain access to binding sites for transcription factors at promoter regions and can restore accessibility for DNA repair factors [9].

The core histones consist of a globular domain and unstructured N-terminal tails which both can be modified by histone-modifying enzymes. Such covalent modifications comprise of acetylation, methylation, phosphorylation, PARylation, and ubiquitylation [10]. To better understand the underlying principles of these chromatin alterations and the involvement of non-coding RNAs in DNA repair, research in the last decade has focused on the contribution of the chromatin environment during genome surveillance. In this review we will cover the role of histone phosphorylation, ubiquitylation, and PARylation of DNA repair factors since these modifications are intimately linked to DNA damage signaling pathways and also play crucial roles for granting access to DNA lesions via remodeling of the chromatin conformation. Furthermore, we will elucidate on novel functions of small non-coding RNAs in the DDR focusing on the events of chromatin that facilitate double-strand break (DSB) repair and nucleotide excision repair (NER).

## 2. Repairing Damaged DNA: NER and DSB Repair

Chromatin reorganization and de-condensation plays a crucial role within all cellular processes which use DNA as their substrate, including transcription, replication, and DNA repair pathways. For instance, one of the several DNA repair pathways relying on conformational changes of chromatin is base excision repair (BER). BER is specifically important in the removal of damaged and altered DNA bases induced by spontaneous hydrolysis of cytosine to uracil, for example, deaminating and alkylating agents or reactive oxygen species released from the cellular respiratory chain. In this review, however, we will focus on the DNA damage response and, further, on double-strand break (DSB) repair and nucleotide excision repair (NER), the DNA repair pathways which handle and remove the most toxic DNA lesions.

Nucleotide excision repair (NER) is an essential DNA repair pathway that counteracts bulky and helix-distorting DNA lesions. The main types of lesions repaired by NER are 6-4 photoproducts and cyclobutane pyrimidine dimers (CPDs), which are caused by exposure to UV light or several chemical agents [11]. NER operates in two sub-pathways, which differ in the nature of lesion recognition. Transcription coupled-NER (TC-NER) operates in transcriptionally-active genes, where stalled Polymerase II elicits the DNA damage response [12]. The global genomic NER branch (GG-NER) deals with lesions in any chromatin environment. In contrast to the TC-NER sub-pathway, in GG-NER DNA lesions are directly recognized by the two damage recognition factors, DDB2 and XPC [13]. After lesion recognition, both sub-pathways converge and lesion verification, unwinding of the DNA, excision of the lesion-containing strand, and refilling of the DNA gap are carried out by the same core machinery. Verification of the DNA lesion is carried out by the repair factor XPA and via the generation of the pre-excision complex, which consists of the TFIIH transcription factor complex with its helicase subunits XPB and XPD. Subsequently, the DNA lesion is excised by the endonucleases XPF and XPG, and the gap is filled by DNA polymerases [12,13]. As detailed below, damage recognition is accompanied by changes in the chromatin conformation and by chromatin signaling pathways that build on phosphorylation, ubiquitylation, and PARylation events. Such chromatin-associated pathways halt the cell cycle and facilitate the DNA damage response (DDR).

Among the most detrimental DNA damages are double-strand breaks (DSBs), which may cause severe genome rearrangements. DSBs are repaired by either error-free homologous recombination (HR) or the error-prone non-homologous end joining (NHEJ) [2,14]. NHEJ involves minimal processing of the damaged DNA and re-ligation of the processed ends. Damage recognition in NHEJ requires the Ku70/80 DNA-binding complex, which binds the broken DNA end and recruits other proteins to facilitate the processing and ligation of the broken ends. Amongst the proteins recruited by Ku to the damage site is DNA-PK, a serine/threonine protein kinase, which induces conformational changes within the damage recognition complex that allows end-processing enzymes to operate on the ends of the double-strand break [15]. DNA-PK also acts in concert with the protein kinase ataxia-telangiectasia, mutated (ATM), and ATM and Rad3-related protein (ATR) to phosphorylate factors that are essential in the DNA damage checkpoint. Further, Ku directly interacts with XRCC4, which recruits specific DNA end processing enzymes to the damage site to generate DNA ends compatible for ligation. Lastly, Ku and XRCC4 associate with the DNA ligase IV complex and localize it to the processed DNA ends for re-ligation [16,17,18]. HR depends on a resection process that involves the generation of single-stranded DNA (ssDNA) intermediates. The ssDNA tails are recognized by RPA, which is then replaced by Rad51. The Rad51-containing nucleoprotein filament searches for, and invades a homologous sequence from the sister chromatid generating a D-loop. After strand invasion DNA polymerase extends the end of the invading strand leading to the formation of holiday junctions. After resolving the intermediates the remaining ssDNA gaps and nicks are repaired by DNA polymerase and DNA ligase. More detailed information on HR repair mechanisms are available in several excellent reviews [19,20]. Sister chromatids and, hence, HR exist only in the S and G2 phases of the cell cycle. In contrast, NHEJ predominates in the G1 phase of the cell cycle. However, how cells choose between HR and NHEJ repair pathways is still elusive [21]. Analogously to NER, chromatin signaling and the regulation of both DSB repair sub-pathways are dependent on phosphorylation, ubiquitylation, and PARylation of factors.

## 3. Phosphorylation Cascades Regulate the DDR

**Figure 1:**
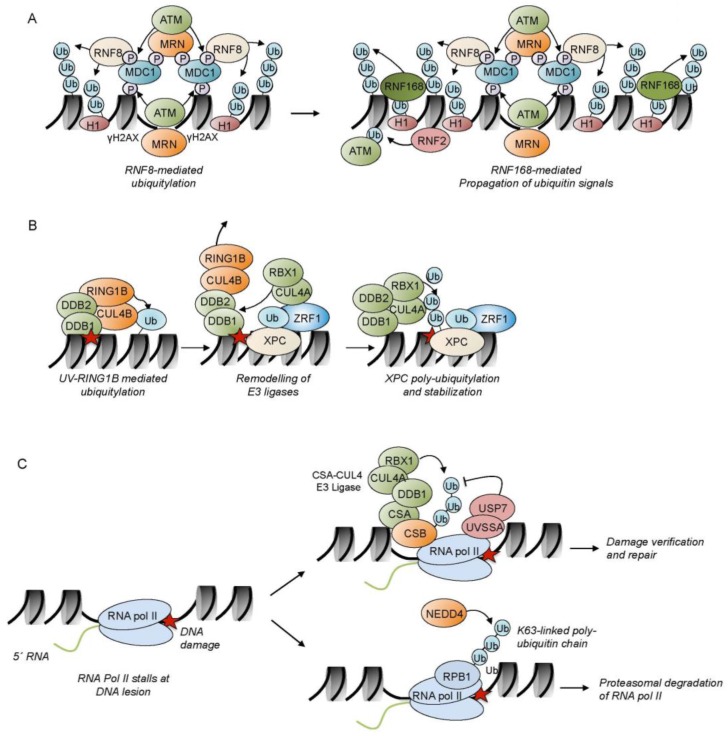
Ubiquitin signaling in both sub-pathways of nucleotide excision repair (NER), global genomic (GG-) and Transcription coupled NER (TC-NER), and double-strand break (DSB) repair. (**A**) Ubiquitin signaling at DSB sites. DSBs are recognized by the MRN (MRE11–RAD50–NBS1) complex, which recruits and activates the ATM kinase through association with its NBS1 subunit. ATM phosphorylates H2AX at S139 (γH2AX), thereby generating a binding platform for MDC1 (mediator of DNA damage checkpoint protein 1). The protein Casein kinase 2 (CK2) (not shown for clarity) constitutively phosphorylates residues within MDC1’s SDT domain. Phosphorylated MDC1 is recognized by NBS1, a subunit of the aforementioned MRN complex. The MDC1-bound MRN subunit NSB1 attracts the ATM kinase, thereby promoting the spreading of γH2AX along the DNA. Phosphorylated MDC1 subsequently recruits the ubiquitin E3 ligase RNF8, which promotes K63-linked poly-ubiquitylation of histone H1 recognized by RNF168. RNF168, in concert with RNF8, ubiquitylates histones H2A and H2AX at K13 and K15 generating recruitment platforms for a range of ubiquitin-binding factors. Additionally, RNF168 can also recognize ubiquitylated forms of H2A, further supporting the efficient spreading of the DNA damage signal along the chromatin. Furthermore, the BMI1-RNF2 (shown as RNF2) module regulates the early initiation steps in the DNA damage response through association with H2AX and stimulating H2AX mono-ubiquitylation which serves as binding platform for ATM; (**B**) The UV-RING1B complex causes mono-ubiquitylation of histone H2A at lysine residue 119 in proximity to the DNA damage site (red star). ZRF1 is localized to the damage site both by interaction with XPC and by binding to H2A-ubiquitin via its ubiquitin binding domain. Furthermore, ZRF1 mediates remodeling of the UV RING1B E3 ligase complex by facilitating the release of the CUL4B-RING1B module and the incorporation of the CUL4A-RBX1 module. The newly-formed DDB-CUL4A E3 ligase complex (green) catalyzes the poly-ubiquitylation of XPC, which is thereby stabilized at the damage site; (**C**) Ubiquitin signaling events during transcription coupled-NER (TC-NER) which either lead to the formation of a pre-incision TC-NER complex (upper part), or mediate proteasomal degradation of RNA pol II (lower part). Elongating RNA polymerase II is stalled after encountering a DNA lesion (indicated as red star). The stalled polymerase serves as a recruiting signal for the Cockayne Syndrome B (CSB) protein. CSB further recruits Cockayne Syndrome WD repeat protein A (CSA) which is part of a multiprotein complex consisting of DNA damage binding protein 1 (DDB1), the scaffold protein CUL4A or CUL4B, respectively, and the E3 ligase RBX1. The removal of CSB initiated by its poly-ubiquitylation is counteracted by the action of the deubiquitinase USP7 which is recruited to stalled RNA pol II by UV-stimulated scaffold protein A (UVSSA) and catalyzes the degradation of the poly-ubiquitin chain on CSB. An alternative pathway initiates subsequent proteasomal degradation of RNA pol II in case TC-NER fails to repair the DNA lesion. The E3 ubiquitin ligase NEDD4 mediates K63 linked poly-ubiquitylation of the RNA pol II component, termed the RNA pol II subunit B1 (RPB1). Poly-ubiquitylated RPB1 is further modified by deubiquitinases and by the elongin A (ELOA)-ELOB-ELOC-CUL5-RING-box protein 2 (RBX2) E3 ligase complex (not shown), which trigger its extraction by the valosin-containing protein (VCP)/p97 ATPase complex followed by proteasomal degradation.

One essential means of signaling during the DNA damage response are phosphorylation events. The damage response at double strand breaks (DSBs) commences with the activation of two specific kinases, the ataxia telangiectasia mutated (ATM) kinase and the ATM and rad3-related (ATR) kinase, which stimulate DNA repair and mediate either apoptosis or checkpoint activation through p53-mediated mechanisms [22]. The main regulator of ATM activation is the MRE11-RAD50-NBS1 (MRN) complex. The MRN complex is one of the first factors which recognize the DSB and plays an essential role in processing of DSBs before repair by either HR or NHEJ is carried out [23]. Notably, PARP1-mediated PARylation events play an important role in the early recruitment of the MRN complex to DSBs highlighting the concerted action, as well as the crosstalk between different post-translational modification processes which mediate chromatin re-organization events [24]. Activation of ATM by the MRN complex causes the phosphorylation of a plethora of factors comprising proteins involved in checkpoint activation such as the checkpoint kinase 2 (CHK2), p53 and DNA-repair proteins, like the breast cancer type 1 susceptibility protein (BRCA1) and the p53 binding protein 1 (53BP1) [1,2]. An important target for the ATM kinase is the histone variant H2A.X (H2AX) (Figure 1A). Phosphorylated H2AX (γH2AX), which occurs early during the DNA damage response, generates a binding platform for the mediator of the DNA damage checkpoint protein 1 (MDC1) [25]. MDC1 is constitutively phosphorylated within its SDT domain by the casein kinase 2 (CK2). This phosphorylation, in turn, creates a binding site for other repair proteins, including the MRN-ATM complex, which brings about the bidirectional spreading of γH2AX for hundreds of kilo-bases along the chromatin fiber emanating from the DSB [26,27]. MDC1 also recruits the ubiquitin E3 ligases RNF8 and RNF168 (Figure 1A), which ubiquitylates histone H2A and the linker histone H1 and facilitates loading of effectors, such as 53BP1 and BRCA1 [28,29]. In contrast to ATM, ATR is activated by a broad spectrum of genomic insults and replication problems [30]. Common to these insults is the generation of single-stranded DNA (ssDNA), which results in the loading of the single-stranded DNA binding protein replication protein A (RPA) [31]. RPA acts as a recruitment platform for ATR via its binding partner ATRIP [32]. ATM and ATR have a plethora of common substrates regulating different cellular functions. Whereas both enzymes for example phosphorylate H2AX, other substrates are predominantly phosphorylated by one specific kinase [22].

It is still largely unresolved how ATR and ATM elicit the damage response upon UV light-induced DNA damage. However, it seems that damage signaling upon UV exposure is dependent on both ATR and ATM. Importantly, H2AX phosphorylation as a major DNA damage marker is predominantly catalyzed by the ATR kinase and occurs in the G1 and S phases of the cell cycle [33,34,35]. However, ATM was also shown to be activated in response to UV-induced DNA damage resulting in phosphorylation of H2AX [36]. Hence, both ATR and ATM are rapidly recruited to DNA lesions following UV-irradiation. Recruitment of both kinases is dependent on the damage recognition factors XPC, DDB2, and XPA only during the G1 phase [37,38]. In contrast, in the S phase, when the UV lesions mainly generate stalled replication forks with long single-stranded DNA, ATR and ATM recruitment to damage loci and subsequent H2AX phosphorylation are likely mediated by different factors. Therefore, UV-induced recruitment of ATR and ATM differ in S and G1 phases owing to the existence of different types of DNA lesions, which facilitate the assembly of different DNA repair factors involved in the process of repair and checkpoint activation.

Taken together, the DNA damage response relies on protein kinase cascades which mediate phosphorylation of DNA repair factors and histones. Phosphorylated substrates operate as binding platforms for effector proteins and regulate the assembly of protein complexes at chromatin. Further, phosphorylation of H2AX is linked to H2A-ubiquitylation and, hence, will likely have an impact on the chromatin conformation. It remains to be seen whether phosphorylation events also crosstalk to other chromatin modifications during the DDR.

## 4. Driving the DDR by Ubiquitylation

Ubiquitin is a small, conserved, regulatory protein that is expressed in almost all tissues of eukaryotic organisms. Ubiquitylation, the attachment of ubiquitin molecules to a substrate protein, can affect the fate and the function of the substrate in various ways. Ubiquitylation of a target protein can trigger its proteasomal degradation; it may alter its cellular localization, affect the enzymatic activity, and facilitate or prevent protein interactions [39,40,41]. An essential histone modification at DNA damage loci is the ubiquitylation of histone H2A, the H2A histone variant H2AX and the linker histone H1 [29,42,43] (Figure 1A). H2A ubiquitylation has been investigated intensively during DSB repair. Ubiquitin-mediated signaling at DSBs is initiated by MDC1-dependent recruitment of the ubiquitin E3 ligase RNF8, which, in concert with Ubc13, catalyzes K63-linked poly-ubiquitylation of histone H1 (Figure 1A) [44]. Ubiquitylated histone H1 subsequently mediates recruitment of the E3 ligase RNF168 to the damage site which, together with RNF8, catalyzes both mono- and poly-ubiquitylation of histones H2A and H2AX at lysine 13–15 [28,42,43,45]. Such ubiquitylated histones also play an essential role in regulating the DSB repair pathway choice by provoking the recruitment of the effector proteins BRCA1 and 53BP1 to the damage site. BRCA1 promotes homologous recombination and is recruited to damaged chromatin through its binding partner within the BRCA1-A complex RAP80, a UBD-containing protein, likely tethering to ubiquitylated histone H2A [46,47,48].

This essential function of BRCA1 in HR is considered one of the major mechanisms contributing to its tumor suppression activity. In contrast, 53BP1 is an essential mediator of NHEJ [49]. Recruitment of 53BP1 to DSBs occurs in a two-pronged fashion and depends on its Tudor domains and on an ubiquitin binding motif [50]. 53BP1 tethers to H4K20 dimethyl marks at damaged chromatin [51,52] and specifically reads RNF168-catalysed H2AK15 ubiquitylation [50]. In parallel to the RNF8-RNF168 pathway, the E3 ligase RING1B catalyzes mono-ubiquitylation of histone H2A at lysine 119 [43,53,54,55], which presumably heterochromatinizes regions surrounding the damage site [56]. Ubiquitylation processes are also a prominent feature of chromatin signalling in NER (Figure 1B). During GG-NER, different E3 ligases were demonstrated to catalyze ubiquitylation of histone H2A. Here, H2A-ubiquitylation is brought about by the E3 ligase RNF8, the UV-DDB-CUL4A/B complexes and the UV-RING1B complex [57,58,59,60,61] (Figure 1B). The UV-RING1B complex, which consists of the subunits DDB1, DDB2, CUL4B, and the E3 ligase RING1B, operates early during DNA damage recognition [61]. It specifically catalyzes the ubiquitylation of lysine 119 of histone H2A, which provides a tethering platform for the H2A-ubiquitin binding protein ZRF1. ZRF1 removes the CUL4B-RBX1 subunits from the UV-RING1B complex and facilitates the incorporation of CUL4A-RBX1, thus mediating the generation of the UV-DDB CUL4A complex at the damage site [61]. The latter catalyzes the poly-ubiquitylation of the DNA damage recognition factor XPC, which stabilizes XPC at chromatin [62]. Hence, the ubiquitylation status of XPC probably acts like a timing device that determines the transition from damage recognition to damage verification [63]. Mono-ubiquitylation at histone H2A is additionally catalyzed by the RING-containing E3 ligase RNF8, which likely does not affect NER directly but rather links it with the DSB-repair pathway [59]. Ubiquitylation events in TC-NER are less well understood and concern poly-ubiquitylation of DNA repair factors rather than ubiquitylation events at chromatin (Figure 1C). In TC-NER, stalled RNA Pol II initiates lesion recognition by recruiting CSB [64]. Notably, CSB, together with DDB1, either one of the scaffold proteins CUL4A or CUL4B, and the E3 ubiquitin ligase RBX1, form another ubiquitin E3 ligase complex. This complex fine-tunes the advance of TC-NER by ubiquitylation of CSB and subsequent proteasomal degradation. CSB ubiquitin chains are cleared by the deubiquitinase USP7, which is recruited to the damage site via the UV-stimulated scaffold protein A (UVSSA) [65,66,67]. UVSSA and USP7 antagonize proteasomal degradation of CSB at the damage site (Figure 1C). In addition, ubiquitylation and degradation of the Pol II subunit RBP1 is a complex two-step process that involves the E3 ubiquitin ligase NEDD4 and the Elongin A ubiquitin ligase [68,69] (Figure 1C). Taken together, predominantly poly-ubiquitylation events participate in the regulation of TC-NER. It still needs to be addressed whether ubiquitylation events at chromatin contribute to TC-NER and whether they provide a binding platform facilitating the recruitment of effector proteins as in GG-NER.

## 5. Impact of Chromatin Remodeling and PARylation on DNA Repair

**Figure 2:**
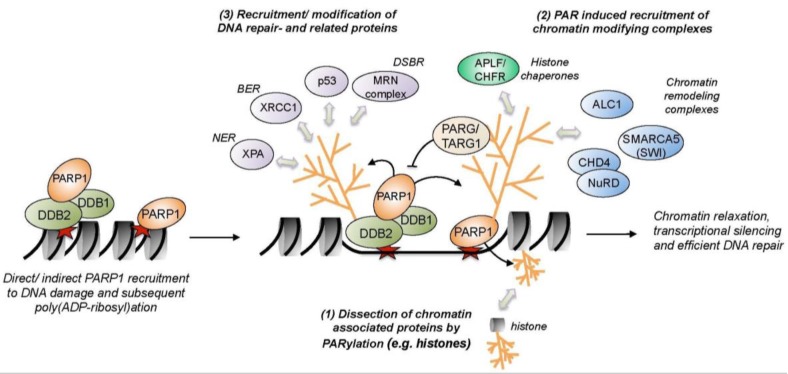
Chromatin re-organization processes mediated by PARylation. Amongst other stimuli, poly (ADP-ribose) polymerases (PARP1) activity is triggered by directly binding to DNA single and double strand breaks (indicated as red star), by post-translational modifications of PARP (e.g., phosphorylation), or by attraction by other DNA damage recognition factors (e.g., DDB2). PARP1-mediated PARylation results in a drastic chromatin re-structuring and decompaction by: (1) dissection of chromatin-associated proteins; (2) stimulation of chromatin re-organization and relaxation by the recruitment of chromatin-modifying complexes; and (3) the recruitment and repair of DNA repair factors. Amongst the proteins recruited to the damage site are the chromatin remodelers amplified in liver cancer 1 (ALC1), SMARCA5, and the NuRD complex, as well as the histone chaperones APLF and CHFR. Furthermore, the PAR-hydolyzing enzmyes poly(ADP-ribose) glycohydrolase (PARG) and terminal ADP-ribose glycohydolase TARG1 are also attracted to chromatin by PAR chains. In addition, PARP1-mediated PARylation plays an important role for the direct recruitment of DNA repair proteins from a wide variety of DNA repair or DDR signaling pathways, such as p53, XRCC1, XPA, and the MRN complex.

PARylation is catalyzed by poly (ADP-ribose) polymerases (PARPs) and constitutes probably one of the earliest post-translational modifications at DNA damage sites. The activity of PARP1 is triggered by DNA damage. PARP generates ADP-ribose polymers by consuming NAD^+^ to modify DNA repair factors and other ancillary proteins involved in DNA repair events and recombination [70,71]. PARP1 elicits the recruitment and regulation of many DNA damage response proteins and has been linked to chromatin remodeling. Remodelers seem to play an essential role during DNA repair, however, the function of many remodelers still remains mysterious. The chromatin surrounding DSBs is rapidly and transiently PARylated (Figure 2) causing the recruitment of the NuRD complex [72,73], which harbors both ATP-dependent chromatin remodeling and histone deacetylase activities. The NuRD subunit CHD4 was found to contain PAR-binding motifs in its amino-terminal region explaining the aforementioned recruitment [74]. At the damage site, NuRD appears to be essential for the localization of DNA repair and checkpoint factors to the damage site and, moreover, it seems to promote transcriptional silencing to support the repair process [75,76] (Figure 2). Likewise, the remodeling ATPase ALC1 (amplified in liver cancer protein 1) that repositions nucleosomes on chromatin is rapidly localized to DSBs by direct interaction with PAR chains on chromatin [77,78]. Only after these PARP1-mediated PARylation events and the following recruitment of the MRN complex and remodeling machineries in response to DSB signaling (Figure 2), ATM activation, and concomitant γH2AX occur. Hence, PARylation at DSBs is a critical event early in the DNA-damage response. Remodeling complexes cause the opening of the condensed chromatin conformation, which allows for the ordered recruitment of DNA repair factors. Chromatin is temporarily locked in a decondensed state, which is also evidenced by the setting of acetylation marks at histones [79,80]. Acetylation, like the spreading of γH2AX, propagates for hundreds of kilo-bases away from the DSB [79,81]. One important acetyltransferase during DSB repair is TIP60, which operates as a subunit of the human NuA4 remodeling complex and catalyzes acetylation of histones H2A, H4, and other DNA repair factors at DSBs [82,83,84]. NuA4 is recruited to chromatin via binding to MDC1, hence, at a later time point than the aforementioned PARylation-dependent remodeling enzymes NuRD and ALC1.

PARylation-mediated chromatin remodeling also plays an essential role within both NER sub-pathways. In GG-NER, the damage recognition factors XPC and DDB2 are targeted by PARP1. PARP1 parylates DDB2 and this causes the recruitment of XPC, thereby promoting efficiency of the NER pathway [71]. PARylation of DDB2 impedes its K48-linked poly-ubiquitylation and subsequent proteasomal degradation and thereby stabilizes DDB2 at the damage site. DDB2 associates with PARP1 and promotes PARylation of chromatin which, in turn, leads to the recruitment of the SWI/SNF chromatin remodeler ALC1 and, hence, chromatin remodeling [85] (Figure 2). Further, both XPC and RAD23B are parylated by PARP1 in response to UV irradiation [86]. XPA, a key factor involved in damage verification within both sub-pathways of NER (Figure 2), shows a high affinity for PAR chains [87], suggesting that PARylation might also play a role at later stages of NER. This non-covalent interaction with PAR chains lowers the DNA binding affinity of XPA. PARP1 also parylates Cockayne syndrome protein CSB after oxidative DNA damage, which inhibits the ATP hydrolysis activity of CSB [88]. CSB itself engages in chromatin remodeling. It remodels nucleosomes in vitro in conjunction with NAP1L1 and NAP1L4 (histone chaperone nucleosome assembly protein 1-like 1/4). CSB also recruits the histone acetyltransferase p300 and the high mobility group nucleosome binding domain-containing protein 1 (HMGN1), further linking lesion recognition in TC-NER to chromatin remodeling [89]. Furthermore, the histone chaperones FACT (facilitates chromatin transcription) [90] and HIRA [13] facilitate transcription restart after lesion-induced transcriptional arrest. Chromatin remodelers from the SWI/SNF family also operate during NER. In baker’s yeast, Rad16 forms part of the NEF4 complex and its SWI/SNF-related ATPase activity is essential for efficient NER [91]. Additionally, NEF4 mediates Gcn5-dependent histone H3 acetylation, which contributes to a decondensed chromatin conformation during the repair process [92]. Further, the damage recognition factors Rad4 (XPC) and Rad23 (RAD23B) associate with the SWI/SNF chromatin remodeling complex subunits Snf6 and Snf5 [93] and the INO80 ATPase or the INO80 complex, respectively, is important for the repair of UV-damaged chromatin likely by enabling the recruitment of XPA and XPC to the damage site [94]. Taken together, chromatin remodeling is an essential feature of DNA damage repair pathways and the loading of nucleosome remodeling complexes on damaged chromatin may be regulated by PARylation of chromatin or DNA repair factors.

## 6. Role of ncRNAs and DICER

**Figure 3:**
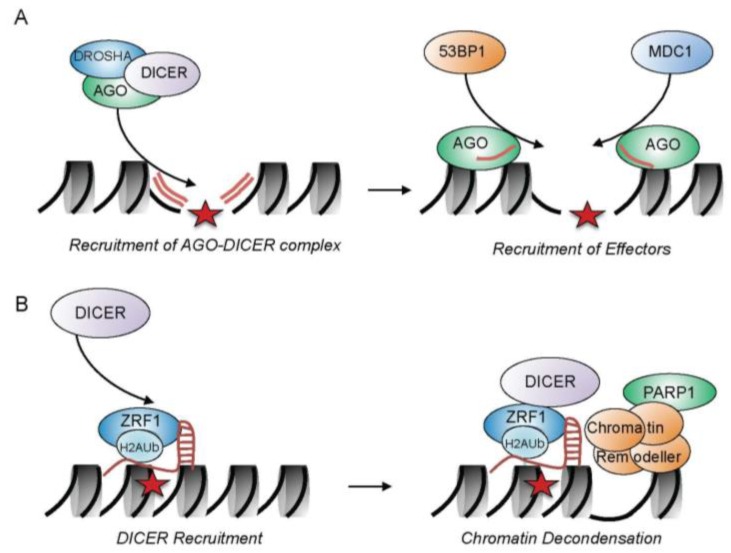
DICER dependent functions in NER and DSB repair. (**A**) Double-stranded RNAs (dsRNAs) are generated from antisense transcripts at the DSB and are subsequently processed by the DROSHA-DICER-AGO complex. AGO-bound ncRNAs localize at the DSB site (indicated as red star) and cause the recruitment of effectors, like 53BP1 and MDC1, by a yet-unknown mechanism; and (**B**) during GG-NER, H2A-ubiquitin-bound ZRF1 provides a binding platform for DICER. Multiprotein complexes consisting of DICER, ZRF1, PARP1 and, potentially, proteins from the SWI/SNF family of remodeling chromatin complexes cause a local decondensation of chromatin.

Small RNAs have emerged as key players in various aspects of biology. Eukaryotes have evolved intricate mechanisms to repair DNA double-strand breaks (DSBs) through well-timed actions of DNA repair factors involving the function of ncRNAs (non-coding RNAs). One factor that processes ncRNAs to control gene expression and genome integrity is the endoribonuclease DICER. DICER chops double-stranded RNA into short RNA fragments, miRNAs, which are crucial to degrade mRNAs in the cytoplasm, and siRNAs, which contribute to the formation of heterochromatin to induce gene silencing [95]. RNA transcribed from a genomic locus is shaped into dsRNA by RDRP (RNA-dependent RNA polymerase) and the resulting dsRNA is concomitantly processed by DICER. siRNAs generated by DICER are assembled into the RITS (RNA-induced transcriptional silencing) complex, which is recruited by chromatin and causes the propagation of H3K9 methylation, a histone mark intimately linked with condensed chromatin states along the chromatin fiber [95]. DICER also contributes to repair of DSBs by generating small ncRNAs which comprise the sequence of the damaged locus [96,97,98,99] (Figure 3A). At DSBs, DICER is of paramount importance for the activation of the DNA damage response and DNA damage checkpoints [96]. Antisense transcripts at the DSB cause the generation of small dsRNAs, which are subsequently processed by the DROSHA-DICER-AGO complex. AGO-bound ncRNAs are localized to the DSB and cause the recruitment of MDC1 and 53BP1 [100]. However, the molecular mechanism of ncRNAs at DSBs is still far from being understood.

Very recently, another role for DICER was identified that deviates from its conventional RNA-dependent function in regulating chromatin conformation [101]. Contrary to its function in generating heterochromatin, DICER also mediates the de-condensation of the chromatin conformation at DNA damage sites during NER (Figure 3B). This unanticipated role for DICER is independent of its ribonuclease activity. DICER is localized to chromatin via the H2A-ubiquitin binding protein ZRF1, which operates during damage lesion recognition in GG-NER. ZRF1 associates to chromatin by both binding to the epigenetic H2A-ubiquitin mark and by interacting with RNA through its SANT domains [102]. ZRF1 and DICER interact with PARP1 and SWI/SNF chromatin remodeling complexes [101], which likely contribute to the decondensation of chromatin structures in GG-NER. Hence, DICER represents a versatile factor that alters the chromatin conformation. Employing its catalytic activity, DICER is involved in chromatin condensation supporting DSB repair in contrast to its contribution to chromatin decondensation in the context of NER where DICER associates with chromatin remodeling factors.

## 7. Concluding Remarks

Chromatin conformation regulates many essential processes, such as replication, transcription, and DNA repair. Hence, components that affect the chromatin state, such as chromatin remodeling complexes and specific chromatin marks, are at the heart of the DNA repair mechanisms. Chromatin marks and modifications of chromatin-associated proteins either cause the recruitment of DNA repair factors or regulate their activity. The interplay of these marks and remodeling activities form the basis of a chromatin signaling network that we are just starting to understand. Future work certainly needs to further address the intricacies and interactions within this chromatin network to better understand how the chromatin environment regulates DNA repair mechanisms.
